# What can we infer about mutation calling by using time‐series mutation accumulation data and a Bayesian Mutation Finder?

**DOI:** 10.1002/ece3.70339

**Published:** 2024-11-10

**Authors:** Takahiro Maruki, April Ozere, Jack Freeman, Melania E. Cristescu

**Affiliations:** ^1^ Department of Biology McGill University Montreal Quebec Canada

**Keywords:** Bayesian Mutation Finder, *Daphnia pulex*, mutation rate, single nucleotide mutations, time‐series mutation accumulation data

## Abstract

Accurate estimates of mutation rates derived from genome‐wide mutation accumulation (MA) data are fundamental to understanding basic evolutionary processes. The rapidly improving high‐throughput sequencing technologies provide unprecedented opportunities to identify single nucleotide mutations across genomes. However, such MA derived data are often difficult to analyze and the performance of the available methods of analysis is not well understood. In this study, we used the existing Bayesian Genotype Caller adapted for MA data that we refer to as Bayesian Mutation Finder (BMF) for identifying single nucleotide mutations while considering the characteristics of the data. We compared the performance of BMF with the widely used Genome Analysis Toolkit (GATK) by applying these two methods to time‐series MA data as well as simulated data. The time‐series data were obtained by propagating *Daphnia pulex* over an average of 188 generations and performing whole‐genome sequencing of 14 MA lines across three time points. The results indicate that BMF enables more accurate identification of single nucleotide mutations than GATK especially when applied to the empirical data. Furthermore, BMF involves the use of fewer parameters and is more computationally efficient than GATK. Both BMF and GATK found surprisingly many candidate mutations that were not confirmed at later time points. We systematically infer causes of the unconfirmed candidate mutations, introduce a framework for estimating mutation rates based on genome‐wide candidate mutations confirmed by subsequent sequencing, and provide an improved mutation rate estimate for *D. pulex*.

## Graphical abstract

Considering the characteristics of mutation accumulation (MA) data, we adapted the existing Bayesian Genotype Caller and we call this Bayesian Mutation Finder (BMF). The performance comparison of BMF and the currently widely used Genome Analysis Toolkit (GATK) using empirical time‐series and simulated MA data indicates that BMF enables more accurate mutation calling than GATK especially when applied to empirical data.

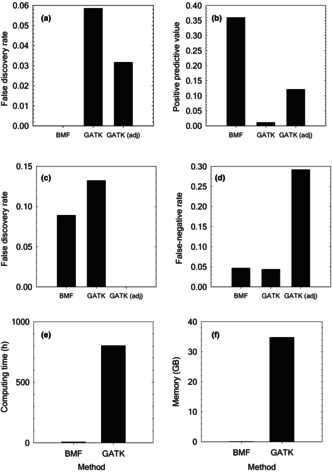

## 1 INTRODUCTION

Mutations generate genetic variation among individuals of natural populations and are essential for the evolution of organisms. Among different types of mutations, single nucleotide mutations (mutations at single nucleotide sites) are the most widely studied, mainly because they are relatively easy to identify, enabling investigation of the genetic mechanisms underlying phenotypic changes and adaptations. Inferring the rates of single nucleotide mutations is essential for evolutionary studies, including population genetics and phylogenetics (Kondrashov, 1988; Loewe & Hill, 2010; Lynch et al., 2016).

To directly investigate spontaneous mutations, researchers often carry out mutation‐accumulation (MA) experiments for many tens of generations. Such MA lines are either descending from few homozygous progenitor strains (typically full‐sib mating or selfing) or from a single progenitor organism when asexual propagation is possible. In all cases, the effective population size of lines is minimized to reduce the effect of natural selection on spontaneous mutations. Thus, the rate of fixation of mutations in such experiments is expected to be close to the mutation rate (Halligan & Keightley, 2009). The correct identification of de novo mutations from the MA data plays a key role in accurately inferring the mutation rate and investigating mechanisms of evolution.

If mutations are correctly identified, MA data using high‐throughput sequencing technologies could provide the most comprehensive picture of spontaneous mutations (Katju & Bergthorsson, 2019; Kondrashov & Kondrashov, 2010). However, accurately identifying de novo mutations from such data is often challenging due to high sequencing error rates, mismapping of sequence reads, variable depths of coverage for sites, individuals, and chromosomes within individuals, as well as uncertainties related to the bioinformatics tools designed to address such inherent problems. Generally, researchers map sequence reads to a reference sequence, call genotypes from mapped sequence reads, filter the genotype calls, and identify candidate mutations. While these steps are relatively straightforward, there is no consensus regarding standardized filtering of candidate mutations (Burda & Konczal, 2023). Researchers take various approaches to identifying candidate mutations and the performance of such procedures is not well understood (Bergeron et al., 2022).

To minimize false discovery, researchers apply various filters when analyzing MA data. Examples of such filters include setting minimum values for the mapping quality score, depth of coverage, base quality score, genotype quality, and nucleotide read count to support each candidate mutation ideally on both strands. However, the effectiveness of such filters is not well understood, and different studies employ various combinations of filters spanning a wide range of parameters. Some filters and/or parameters can be too strict or too relaxed, resulting in either high false‐negative rates or high false‐positive rates.

After identifying candidate mutations, many studies examine mapped sequence reads covering the candidate mutations using the Integrative Genomics Viewer (IGV) (Thorvaldsdottir et al., 2013) and validate a subset of the candidate mutations using Sanger sequencing (e.g., Behringer & Hall, 2016; Keightley et al., 2009, 2014; Long et al., 2016). Although such approaches are very useful, they are not always practical. For example, identifying signatures of mismapping using the IGV inspection requires manual examination of mapped sequence reads around each candidate mutation in both the mutant and other MA lines (Flynn et al., 2017; Ho et al., 2020; Keightley et al., 2014). Validating the candidate mutations using Sanger sequencing is equally challenging when the numbers of candidate mutations and analyzed MA lines are relatively large. Thus, it is generally difficult to estimate the validity of candidate mutations and minimize both false positives and false negatives. The reported false discovery rates of mutation identification based on Sanger sequencing vary widely with some studies finding zero false‐positive mutation (e.g., Behringer & Hall, 2016; Ho et al., 2020; Keightley et al., 2009), and others reporting high false discovery rates even when employing strict filtering procedures (e.g., Burda & Konczal, 2023; Flynn et al., 2017; Keightley et al., 2014; Long et al., 2016). Relatively few studies estimated the false‐negative rate of mutation identification, and the effect of filtering procedures on the false‐negative rate is poorly understood (but see Behringer & Hall, 2016; Keightley et al., 2014; Konrad et al., 2019).

Long‐term MA experiments typically involve model organisms with short generation time such as *Caenorhabditis elegans*, *Daphnia pulex*, *Drosophila melanogaster*, and *Saccharomyces cerevisiae*. The freshwater microcrustacean *D*. *pulex* is particularly well‐suited for MA studies due to its short generation time (10–12 days), the availability of reference genomes (TCO; Colbourne et al., 2011, PA42; Ye et al., 2017, D8.4A; Barnard‐Kubow et al., 2022, KAP4), and the reproductive strategy that allows for clonal propagation. While daphniids typically reproduce by cyclical parthenogenesis, an alternation between sexual and asexual reproduction, the North American *D. pulex* has a more unusual reproductive strategy. While some lineages retain the ancestral cyclical parthenogenetic strategy, many lineages of *D. pulex* have completely lost the ability to reproduce sexually and are obligate parthenogens (Hebert & Crease, 1980, 1983; Innes & Hebert, 1988). Previous MA studies found that mutation rate estimates derived from obligatory parthenogenetic isolates are comparable to those derived from cyclically parthenogenetic isolates (Keith et al., 2016; Xu et al., 2012). Estimates of single nucleotide mutation rates in previous studies (Bull et al., 2019; Flynn et al., 2017; Keith et al., 2016, 2021) ranged from 1.57 × 10^−9^ (Keith et al., 2021) to 3.8 × 10^−9^ (Keith et al., 2016) per site per generation per line.

In this study, we adapted the existing Bayesian Genotype Caller (BGC) (Maruki & Lynch, 2017) for MA data and we call it the Bayesian Mutation Finder (BMF). We assess its performance by comparing it with the well‐established Genome Analysis Toolkit (GATK) (DePristo et al., 2011; McKenna et al., 2010; Van der Auwera et al., 2013) and by applying it to time‐series mutation accumulation (MA) data of *D. pulex* generated with high‐throughput sequencing and moderate depths of coverage (mean 10–14×).

To assess the performance when true mutations are known, we also applied BMF to simulated data generated from the reference sequence of *D. pulex* used in the empirical data analysis. As true mutations are expected to be shared between time points exclusively within individual MA lines, the positive predictive value and false discovery rate were estimated from the number of shared candidate mutations across MA lines and time points. Furthermore, we introduce a framework for estimating mutation rates based on genome‐wide candidate mutations confirmed by subsequent sequencing and provide an improved mutation rate estimate for *D. pulex*.

## 2 MATERIALS AND METHODS

### 2.1 Analyzed data

In this study, we analyzed MA in 14 MA lines across three time points (Table S1) spanning an average of 188 generations. The number of generations of the 14 analyzed MA lines at time points one, two, and three ranged from 73 to 88, from 130 to 174, and from 161 to 204, respectively. The mean generation numbers were 82, 159, and 188 at time points one, two, and three, respectively. The MA lines were independently propagated from a single obligate asexual isolate of the freshwater microcrustacean *Daphnia pulex* collected from Canard Pond (42° 12, −82° 98) in Windsor, ON, Canada. Therefore, given the generally low mutation rates and strictly asexual propagation, each de novo mutation is expected to be found in a heterozygous genotype at a focal locus in only one of the MA lines. The effective population size of the MA lines was minimized by transferring a single neonate to fresh media every generation in each line to minimize the confounding effect of natural selection on mutation rate estimates. The MA data at the first time point were sequenced by Flynn et al. (2017) and are available at the NCBI SRA (BioProject ID PRJNA341529). In addition to the previously published data, we sequenced all 14 MA lines at two subsequent time points, and the data are also available at the NCBI SRA (BioProject ID PRJNA847774).

### 2.2 Sample preparation and sequencing for the subsequent time points

The samples of 14 MA lines at subsequent time points were prepared and sequenced as in Flynn et al. (2017). To minimize sequencing other organisms, including bacteria and algae, adult animals were placed in an antibiotics solution and fed with sterile beads prior to the tissue collection (Fields et al., 2015). DNA was extracted from the adult animals using the cetyltrimethylammonium bromide method (Doyle & Doyle, 1987). Libraries were prepared with a tagmentation‐based method, tagging MA lines with dual indexes and using a modified transposase enzyme (Adey et al., 2010), following a modified protocol for the Illumina Nextera kit (Baym et al., 2015). A pooled sample of the tagged MA lines was sequenced once with one lane using the Illumina HiSeq X and HiSeq X Ten platforms at time points two and three, respectively, with 150 bp paired‐end reads at the Genome Quebec Innovation Center at McGill University. It is important to note that a different platform (Illumina HiSeq 2000) and read length (100 bp) were used at time point one (Flynn et al., 2017).

### 2.3 Data processing

To make a fair comparison of the performance of BMF and GATK, we processed FASTQ files of raw sequence reads for subsequent analyses in the same way. The FASTQ files of the sequence reads were processed by various filtering procedures to ensure high‐quality data (Text S1). First, adapter sequences were trimmed from the sequence reads using Trimmomatic, version 0.39 (Bolger et al., 2014). The adapter‐trimmed sequence reads were mapped to the reference sequence of *D. pulex* (PA42, version 4.2, available at the NCBI GenBank, BioProject ID PRJEB46221, Ye et al., 2021) concatenated with the mitochondrial DNA sequence of *D. pulex* (Crease, 1999), available at the NCBI GenBank, BioProject ID PRJNA11866 using BWA‐MEM (version 0.7.17; https://arxiv.org/abs/1303.3997). The size of the reference sequence is 155.1 Mb (Ye et al., 2021). The percentage of sequence reads mapped to the reference sequence in each MA line was derived from each corresponding BAM file of mapped sequence reads using the flagstat function of Samtools, version 1.11 (Li et al., 2009). BAM files were filtered using Samtools. We retained only properly paired reads with at least 20 mapping‐quality scores and removed unmapped reads, non‐primary reads, reads that failed platform/vendor quality checks, PCR or optical duplicate reads, and supplementary reads. Duplicate reads were marked using GATK, version 4.1.9.0 (DePristo et al., 2011; McKenna et al., 2010; Van der Auwera et al., 2013). Overlapping read pairs were clipped using BamUtil, version 1.0.14 (http://genome.sph.umich.edu/wiki/BamUtil). Reads that mapped to the mitochondrial DNA were excluded using Samtools.

### 2.4 Genotype calling using BMF

We adapted the existing BGC (Maruki & Lynch, 2017) for MA data to improve genotype calling at single nucleotide sites from MA data with moderate depths of coverage, and we call this BMF (https://github.com/Takahiro‐Maruki/BMF). BMF calls genotypes taking into consideration the characteristics of MA data. These characteristics include (1) generally low mutation rates such that each de novo mutation appears in only one of the MA lines; (2) independent evolution of MA lines derived from a single ancestral line, such that MA lines initiated from the ancestral line should have the same genotype at a locus unless mutation occurs. BMF calls genotypes using BGC, which improves the accuracy of genotype calls from high‐throughput sequencing data with limited coverage by incorporating the sample‐level information on genotype frequencies and error rates estimated beforehand by the genotype frequency estimator (GFE) (Maruki & Lynch, 2015) using Bayes' theorem. The error rate is estimated from the nucleotide read counts at each site, where errors include not only those in sequencing but also in sample preparation, library preparation, and read mapping. The error rate estimated specifically for each site is especially useful for identifying potentially problematic sites and testing the goodness of fit between the observed nucleotide read counts and their binomial expectation. BGC performs as well as and in many cases better compared to other widely used methods (Maruki & Lynch, 2017) and is generally applicable to any sample of diploid organisms. Incorporating the sample‐level information in calling individual genotypes is especially useful in MA data, where MA lines derived from a single progenitor individual are expected to have the same genotype at each locus without mutation. Furthermore, single nucleotide polymorphisms (SNPs) are identified in a rigorous statistical framework beforehand by the GFE (Maruki & Lynch, 2015) in BMF, which is important for avoiding analyzing false SNPs. GFE and BGC assume at most two alleles at each site and sites containing more than two alleles are identified by the high‐coverage genotype caller (HGC) (Maruki & Lynch, 2017) and removed from subsequent analyses. The ancestral genotype is inferred as the genotype most abundant (consensus genotype) in the sample at each site. The BGC genotype calls at bi‐allelic sites are filtered by a two‐tailed binomial test (McDonald, 2014), which examines the goodness of fit between the observed nucleotide read counts and expected nucleotide read counts under the binomial distribution, given a fixed number of nuclear read counts (see also Flynn et al. (2017) for a previous similar approach). The null hypothesis of the binomial test is that nucleotide reads in a diploid MA line with a genotype call at a given site were equally sequenced from the two chromosomes. The two‐tailed binomial test calculates the probability of nucleotide read counts deviating from the binomial expectation as large as or greater than the observed values under the null hypothesis using the method of small *p* values and it is conservative. The probability is first calculated assuming that the genotype of the MA line is the same as the inferred ancestral genotype at the site. When the inferred ancestral genotype is heterozygous containing alleles *A* and *a*, the probability of the observed counts of alleles *A* and *a*, *n*_*A*_ and *n*_*a*_, is calculated as

(1) $$P\left(n_{A},n_{a}|\;\mathrm{Aa}\;\right)=\frac{\left(n_{A}+n_{a}\right)!}{n_{A}!n_{a}!}{\left(\frac{1}{2}-\frac{\hat{\varepsilon }}{3}\right)}^{n_{A}}{\left(\frac{1}{2}-\frac{\hat{\varepsilon }}{3}\right)}^{n_{a}}$$

The *p*‐value for the binomial test is calculated as

(2) $$p=\sum_{i,j}P\left(i,j|\mathrm{Aa}\right),$$

where the summation is taken over a set of integers *i* and *j* such that *P*(*i*, *j*|*Aa*) ≤ *P*(*n*_*A*_, *n*_*a*_|*Aa*) and *i* + *j* = *n*_*A*_ + *n*_*a*_. If the null hypothesis with the heterozygous inferred ancestral genotype is rejected, the genotype call is deemed homozygous only when all reads in the MA line consists of one nucleotide. When the inferred ancestral genotype is homozygous containing allele *A*, the probability of the observed counts of alleles *A* and *a*, *n*_*A*_ and *n*_*a*_, is

(3) $$P\left(n_{A},n_{a}|\mathrm{AA}\right)=\frac{\left(n_{A}+n_{a}\right)!}{n_{A}!n_{a}!}{\left(1-\frac{\hat{\varepsilon }}{3}\right)}^{n_{A}}{\left(\frac{\hat{\varepsilon }}{3}\right)}^{n_{a}},$$

where $\hat{\epsilon }$ is the error rate estimate at the site by GFE (Maruki & Lynch, 2015).

The *p*‐value for the binomial test is calculated as

(4) $$p=\sum_{i,j}P\left(i,j|\mathrm{AA}\right),$$

where the summation is taken over a set of integers *i* and *j* such that *P*(*i*, *j*|*AA*) ≤ *P*(*n*_*A*_, *n*_*a*_|*AA*) and *i* + *j* = *n*_*A*_ + *n*_*a*_. If the null hypothesis with the homozygous inferred ancestral genotype is rejected, an additional test using binomial expectation given a heterozygous genotype (Equation 2) is carried out, where the genotype call is deemed heterozygous only when the null hypothesis with the heterozygous genotype is not rejected and both nucleotides are supported by at least two reads. Because we apply the binomial test to a particular genotype in a particular individual and the test is conservative, multiple‐testing correction is not necessary.

To prepare an input pro file, where counts of nucleotides A, C, G, and T separated by slashes (*n*_*A*_/*n*_*C*_/*n*_*G*_/*n*_*T*_) are shown at each site in each MA line, an mpileup file of per‐site sequence read data in the 14 MA lines was made from the processed BAM files at each time point using Samtools. Then, the pro file of nucleotide read counts was made from the mpileup file at each time point using mpileup2pro (https://github.com/Takahiro‐Maruki/mpileup2pro). Genotypes of the MA lines were called from the pro file using BMF. The significance levels of the binomial test given heterozygous and homozygous genotypes were set at 0.025 and 0.05, respectively. We set the significance level for heterozygous genotypes at half the value (0.025) of that for homozygous genotypes (0.05) because the observed nucleotide read counts in heterozygous genotypes are more likely to deviate by chance from the expected nucleotide read counts. The full procedure used to call single nucleotide mutations from the MA data using BMF, which can be applied to other MA data, is shown in Text S2.

### 2.5 Genotype calling using GATK

To examine how BMF compares with the currently widely used GATK (DePristo et al., 2011; McKenna et al., 2010; Van der Auwera et al., 2013), we called corresponding genotypes using GATK (version 4.1.9.0) (Text S3). Genotypes of each MA line were called using HaplotypeCaller from the processed BAM file. The genotype calls were refined by joint genotype calling incorporating sample‐level information using GenotypeGVCFs. SNPs were extracted from the VCF file of genotype calls using SelectVariants, excluding sites involved in putative repetitive regions. The genotype calls involved in SNPs were filtered using VariantFiltration with generally recommended parameter cut‐off values (QD < 2.0, QUAL < 30.0, SOR > 3.0, FS > 60.0, MQ < 40.0, MQRankSum < −12.5, and ReadPosRankSum < −8.0). To examine whether the generally recommended parameter cut‐off values in hard filtering were suitable for our data, we made plots of the distribution of the parameter values (Figure S2) and chose their adjusted cut‐off values (QD < 11, QUAL < 30, SOR > 2, FS > 10, MQ < 41, MQRankSum < −2.5, and ReadPosRankSum < −2.5) such that SNPs with parameter values indicating low quality are removed from subsequent analyses. Moreover, to examine the effect of chosen parameter cut‐off values on the performance of GATK hard filtering, we used another set of parameter cut‐off values (QD < 10, QUAL < 30, SOR > 3, FS > 60, MQ < 40, MQRankSum < −2, and ReadPosRankSum < −3.5), based on the histograms of the parameter values and cut‐off values used in published studies (Table S2; Text S4). To find whether the low rates of within‐line sharing of candidate mutations by both methods and better performance of BMF than GATK were caused by requiring too many (eight) nucleotide reads to call genotypes with moderate depths of coverage, we carried out corresponding performance comparison requiring at least six nucleotide reads to call genotypes.

### 2.6 Removal of potentially problematic sites

To avoid identifying false mutations, we removed potentially problematic sites. In the results using BMF, tri‐ and tetra‐allelic sites were identified using HGC (Maruki & Lynch, 2017) requiring at least six nucleotide reads to call individual genotypes at each site and were removed from subsequent analyses. Sites with total coverage (the sum of depths of coverage over the MA lines) deviating from typical values, those with error‐rate estimates >0.01, and those involved in putative repetitive regions identified by RepeatMasker (http://www.repeatmasker.org) were also removed from subsequent analyses. The corresponding filtering procedures except that based on error‐rate estimates were applied in the results using GATK.

### 2.7 Mutation identification

Mutations were identified from the genotype calls of all 14 MA lines at each site and each time point using BMF and GATK. To avoid identifying mutations resulting from reference bias, mutations were identified only at sites where the inferred ancestral genotype was homozygous for the reference nucleotide. To minimize uncertainties resulting from missing data and low coverage, mutations were identified only at sites where all 14 MA lines had genotypes called from at least eight nucleotide reads. Requiring a minimum depth of coverage to call individual genotypes is important for distinguishing true heterozygous genotype calls from erroneous ones (Maruki & Lynch, 2017). The genotype call in each MA line was compared to the inferred ancestral genotype at each site and mutant nucleotides were identified as those not found in the inferred ancestral genotype. Only mutations found exclusively in one of the 14 MA lines at a given site were kept for subsequent analyses.

### 2.8 Assessment of candidate mutations

As true mutations are expected to be shared within MA lines between time points but not shared between MA lines, we counted candidate mutations shared among MA lines and time points to assess their overall quality. Using the BMF and GATK counts, we calculated the rates of candidate mutations shared between different MA lines (cross‐line sharing) and that of candidate mutations shared between time points within MA lines (within‐line sharing). The cross‐line sharing rate was calculated by dividing the number of candidate mutations shared between different MA lines by the total number of candidate mutations at an earlier time point (time point one or two) callable (passed the filters and had genotype calls in all 14 MA lines with the inferred ancestral genotype homozygous for the reference nucleotide) at a later time point (time point two or three) as a function of time‐point pairs. Similarly, the within‐line sharing rate was calculated by dividing the number of candidate mutations consistently found within MA lines at both time points by the total number of candidate mutations at an earlier time point callable at a later time point as a function of time‐point pairs. The rates of cross‐line and within‐line sharing of candidate mutations were used as proxies for the false discovery rate and positive predictive value, respectively, to assess the performance of the two methods.

To examine the consistency of mutation identification between time points, the rate of unconfirmed candidate mutations at a time point as a function of the time point (time point one or two) when the candidate mutation was first identified was calculated conditioned on genotype calls at both time points in the results using BMF. To investigate the cause of candidate mutations unconfirmed at later time points, we examined the nucleotide read counts underlying BMF genotype calls across time points.

### 2.9 Estimation of the mutation rate

Using the candidate mutations identified with BMF, we estimated the mutation rate *μ* per site per generation per MA line at each time point. As in Flynn et al. (2017) and Bull et al. (2019), *μ* was calculated as $\mu =\frac{m}{2\mathrm{NTg}}$, where *m*, *N*, *T*, and *g* denote the total number of candidate mutations, number of callable sites, total number of analyzed lines (14 in this study), and the mean number of generations, respectively (Keith et al., 2016). The standard error of the mean of *μ* was estimated as $\sqrt{\frac{\mu }{2\mathrm{NTg}}}$, applying the Poisson distribution, as in Keith et al. (2016). To examine mutation bias, we calculated mutation rate estimates conditional on the inferred ancestral nucleotide, pooling data across three time points. The ratio of transition mutation rate to transversion mutation rate estimates (Ts/Tv) and AT bias were calculated by dividing the transition mutation rate estimates by transversion mutation rate estimates and dividing the estimates of G/C to A/T mutations by those of A/T to G/C mutations, respectively. Given the high rate of unconfirmed candidate mutations, mutation rates were also estimated for pairs of time points using sites callable at both time points, where the mutation rate for time‐point pair (*i*,*j*), $\mu_{\left(i,j\right)}$, was calculated as $\mu_{\left(i,j\right)}=\frac{m_{\left(i,j\right)}}{2N_{\left(i,j\right)\mathrm{Tg}}}$, where *m*_(*i*,*j*)_ and *N*_(*i*,*j*)_ denote the total number of candidate mutations found at time points *i* and *j* and number of sites callable at time points *i* and *j*, respectively. The standard error of the mean of $\mu_{\left(i,j\right)}$ was estimated as $\sqrt{\frac{\mu_{\left(i,j\right)}}{2N_{\left(i,j\right)}\mathrm{Tg}}}$.

### 2.10 Performance comparison of BMF and GATK with simulated data

To compare the performance of BMF and GATK when true mutations are known, we generated sequence reads from independently evolving 14 diploid individuals using PA42.4.2 and NEAT (Stephens et al., 2016) under the default mutation and sequencing models and applied both methods to the simulated data. Specifically, paired‐end sequence reads with the mean 10× depth of coverage and 150 bp length were independently generated in each of the 14 individuals using the following command:

gen_reads.py ‐r PA42.4.2.fasta ‐R 150 ‐M 0.0001 ‐o MAL01_PA42.4.2 ‐‐bam ‐‐vcf ‐‐pe 300 30

The simulated sequence reads were mapped to PA42.4.2 and processed in the same way as in the empirical data set. Moreover, we applied the same filters as in the empirical data set, setting the minimum and maximum cut‐off values for the total depth of coverage (sum of depths of coverage over the MA lines) at 70 and 170, respectively. Candidate single nucleotide mutations identified by BMF and GATK using hard filtering with generally recommended parameter cut‐off values were compared to the true single nucleotide mutations. The false discovery rate and false‐negative rate were, respectively, calculated as the fraction of false positives among candidate single nucleotide mutations and fraction of false negatives among true single nucleotide mutations at callable sites. As in the empirical data set, corresponding GATK results were obtained using adjusted cut‐off values (QD < 10, QUAL < 30, SOR > 2, FS > 10, MQ < 40, MQRankSum < −2, and ReadPosRankSum < −3.5) in hard filtering.

To compare the performance of BMF and GATK when depths of coverage are higher, we generated corresponding simulated MA data specifying the mean 30× depth of coverage, by adding “‐c 30” to the above command. Both methods were applied to the simulated data, setting the minimum and maximum coverage cut‐off values for the total depth of coverage at 200 and 460, respectively. The corresponding GATK results were similarly obtained using adjusted cut‐off values (QD < 10, QUAL < 30, SOR > 3, FS > 20, MQ < 40, MQRankSum < −2.5, and ReadPosRankSum < −3) in hard filtering.

## 3 RESULTS

### 3.1 Analyzed data

The percentage of sequence reads mapped to the reference sequence was generally high (95%–97%). The mean depths of coverage per site per *Daphnia pulex* MA line at time points one, two, and three were 10.8, 13.8, and 10.0, respectively (Table 1). Small fractions (0.16%, 0.19%, and 0.18% at time points one, two, and three, respectively) of the significantly (at the 5% level) polymorphic sites were identified as tri‐ and tetra‐allelic sites by HGC (Maruki & Lynch, 2017) and removed from subsequent analyses. The filters based on total coverage (the sum of depths of coverage over the MA lines), error‐rate estimates, and involvement in putative repetitive regions, respectively removed similarly small fractions (0.21%–0.52%, 2.01%–3.36%, and 4.98%–6.46%) of significantly polymorphic sites.

**TABLE 1:** Depths of coverage in the analyzed data.

Time point	Mean generations	Read length (bp)	Total coverage	Mean coverage
1	82	100	151.3 [40, 400]	10.8
2	159	150	193.6 [40, 500]	13.8
3	188	150	140.4 [40, 400]	10.0

*Note*: The mean of the total coverage (sum of depths of coverage over the MA lines) as a function of the time point is shown. The numbers in the square brackets denote the minimum and maximum of the total coverage of the analyzed sites. The mean coverage per MA line, which was calculated by dividing the mean total coverage by 14, as a function of the time point is also shown.

### 3.2 Mutation identification

Consistent with previous studies (Bull et al., 2019; Flynn et al., 2017), small numbers (9–64) of candidate mutations were identified by BMF in an MA line at a given time point (Table S3). There was no significant correlation (*p* > .05) between the mean depth of coverage and number of candidate mutations in MA lines (Figure S1), which is also consistent with the previous findings by Flynn et al. (2017), indicating that coverage is not the main factor limiting the identification of candidate mutations at callable sites.

### 3.3 Assessment of candidate mutations

**FIGURE 1:**
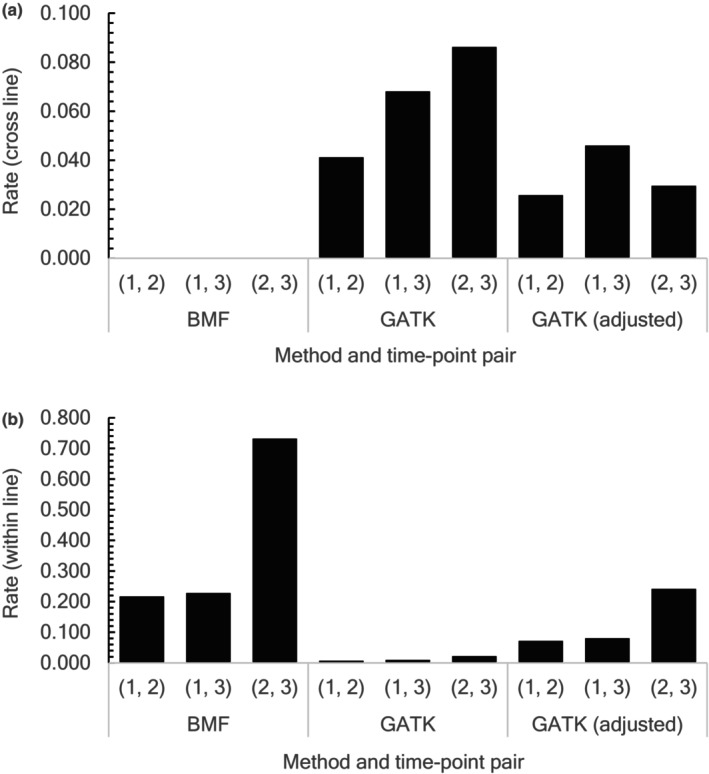
Rates of cross‐line (a) and within‐line (b) sharing of candidate mutations identified using BMF, GATK with generally recommended parameter cut‐off values in hard filtering, and GATK with adjusted parameter cut‐off values in hard filtering.

To examine how the candidate mutations are shared across MA lines and time points, we made a matrix of the number of candidate mutations shared between pairs of MA lines across time points (Table S4). The rate of cross‐line sharing of candidate mutations identified by BMF was zero (Figure 1a), indicating that the false discovery rate of mutation identification by BMF is low. The rates of within‐line sharing of candidate mutations for time‐point pairs (1, 2), (1, 3), and (2, 3) were 0.22, 0.23, and 0.73, respectively (Figure 1b). The rates of candidate mutations first identified at time point one and unconfirmed at time points two and three were rather high, 0.78 and 0.73, respectively. The rate of candidate mutations first identified at time point two and unconfirmed at time point three was 0.16. These results show that many candidate mutations, especially those first identified at time point one, were unconfirmed at later time points. Examination of nucleotide read counts underlying the genotype calls (Table S5) revealed that the candidate mutations unconfirmed at later time points were mainly due to the absence of the mutant nucleotide read in the MA line at later time points. To determine if the mutant nucleotide read can be found in the MA line at later time points at sites with unconfirmed candidate mutations by relaxing the filtering procedure taken in the data preparation process, we prepared data including supplementary reads (those involved in chimeric alignments) and identified candidate mutations. Including supplementary reads did not change the rates of cross‐line sharing and within‐line sharing of candidate mutations. To further examine the effect of the filtering procedure, we calculated the cross‐line and within‐line sharing of candidate mutations using data before filtering. As expected, in the absence of filtering, the rates of within‐line sharing of candidate mutations substantially decreased (0.13, 0.15, and 0.12), for time‐point pairs (1, 2), (1, 3), and (2, 3), respectively, and rates of cross‐line sharing of candidate mutations remained largely unchanged (0.00, 0.00, and 0.00, for time‐point pairs (1, 2), (1, 3), and (2, 3), respectively). Thus, filtering results in clear improvements (increase in the rates of within‐line sharing of candidate mutations) mainly due to the removal of duplicate sequence reads, as the rates using data before removing duplicate sequence reads were similar to those using data before filtering.

### 3.4 Estimation of the mutation rate

**FIGURE 2:**
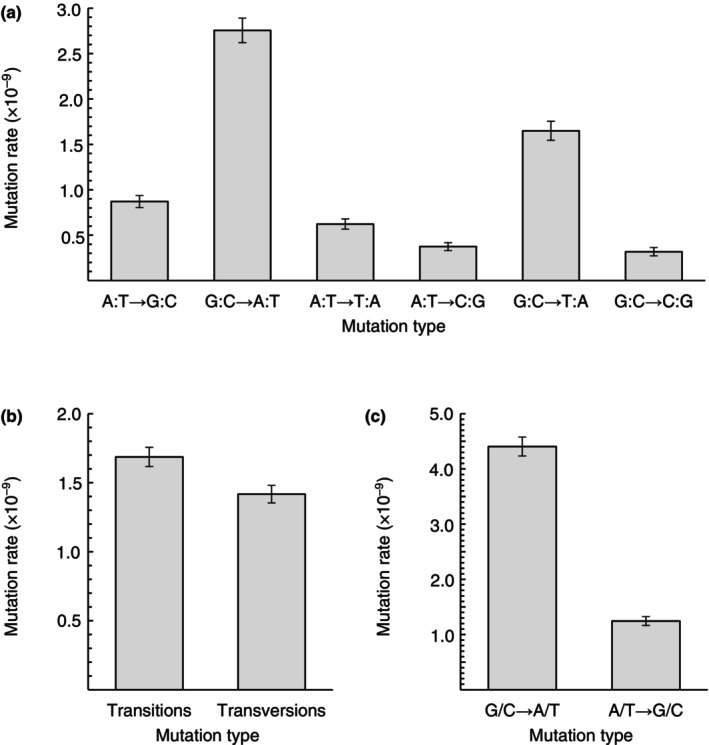
Conditional mutation rate estimates. Rate estimates conditional on the inferred ancestral nucleotide in pooled data across three time points for each mutation type (a), transitions and transversions (b), and G/C → A/T and A/T → G/C mutations (c) are shown. Error bars denote the standard error of the mean.

Using the identified candidate mutations, we estimated the mutation rate per site per generation per MA line at each time point (Table 2a). The estimated mutation rates at time points one, two, and three were 6.44 × 10^−9^ ± 3.82 × 10^−10^, 2.22 × 10^−9^ ± 9.85 × 10^−11^, and 3.36 × 10^−9^ ± 1.98 × 10^−10^ per site per generation per line, respectively, where the standard error of the mean is also shown. Consistent with previous studies (Bull et al., 2019; Flynn et al., 2017; Keith et al., 2016), mutation rate estimates conditional on the inferred ancestral nucleotide (Figure 2a) showed transitional mutation bias (Figure 2b) and AT bias (Figure 2c). The ratio of transitional to transversional mutation rate estimates (Ts/Tv) was 1.19, which was more than twice compared to the null expectation of 0.5 if nucleotides have equal probability of mutating to any other nucleotide. The estimated rate of G/C → A/T mutations was 3.54 times higher than that of A/T → G/C mutations (where G/C → A/T includes both G:C → A:T and G:C → T:A mutations, and A/T → G/C includes both A:T → G:C and A:T → C:G mutations). Given the high rates of unconfirmed candidate mutations, we also estimated mutation rates for pairs of time points using sites callable at both time points (Table 2b). The mutation rate estimates for time‐point pairs (1, 2), (1, 3), and (2, 3) were 1.01 × 10^−9^ ± 1.62 × 10^−10^, 9.00 × 10^−10^ ± 2.12 × 10^−10^, and 1.10 × 10^−9^ ± 1.30 × 10^−10^ per site per generation per line, respectively.

**TABLE 2:** Information on the values used for the entries in the equations for estimating the A) mutation rate at each time point and B) mutation rate for each time‐point pair.

A				
Time point	Total number of candidate mutations	Number of callable sites	Fraction of callable sites in PA42.4.2	Mean number of generations
1	284	19,251,067	0.12	82
2	510	51,518,237	0.33	159
3	288	16,237,913	0.10	188

B				
Time‐point pair	Total number of confirmed candidate mutations	Number of sites callable at both time points	Fraction of sites callable at both time points in PA42.4.2	Mean number of generations
(1, 2)	39	16,816,789	0.11	82
(1, 3)	18	8,734,340	0.06	82
(2, 3)	71	14,564,261	0.09	159

*Note*: The total number of analyzed lines is 14 at all three time points.

### 3.5 Comparison of BMF and GATK results

**FIGURE 3:**
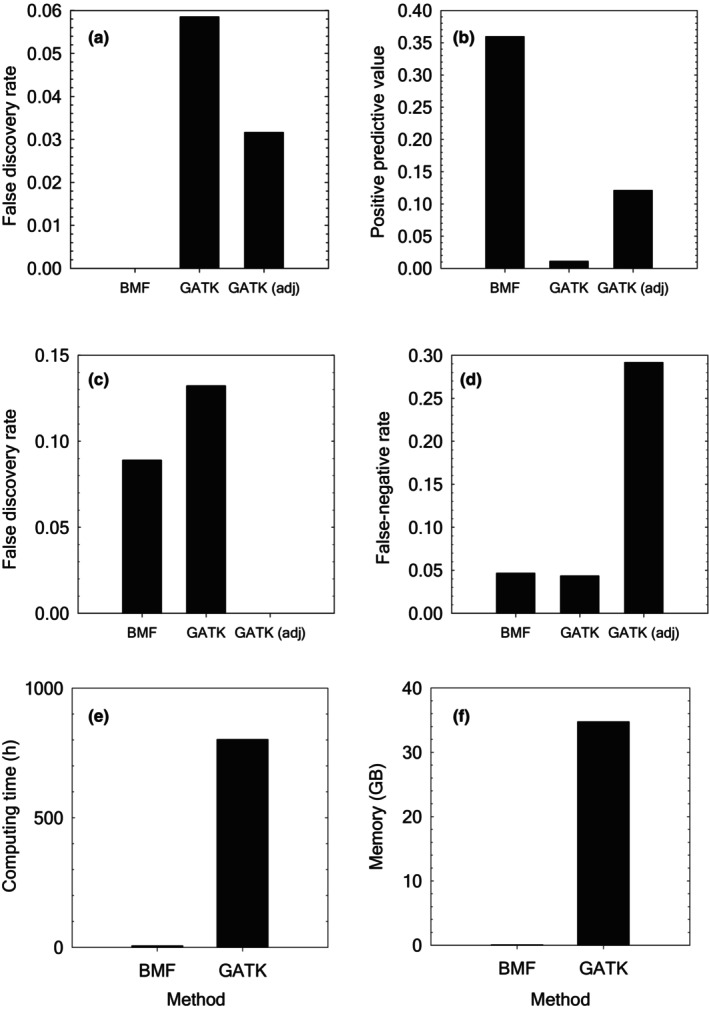
Performance comparison of BMF and GATK. Performance of BMF is compared to that of GATK in terms of (a) false discovery rate with empirical data, (b) positive predictive value with empirical data, (c) false discovery rate with simulated data with moderate depths of coverage, (d) false‐negative rate with simulated data with moderate depths of coverage, (e) computing time (in hours), and (f) memory consumption (in GB). The false discovery rate and positive predictive value with empirical data were calculated with pooled data across time point pairs. The computing time and memory are those taken for analyzing data of 14 MA lines at time point one. GATK results with generally recommended parameter cut‐off values and adjusted cut‐off values in hard filtering are shown in a, b, c, and d.

In stark contrast with the results using BMF, the results using GATK (with generally recommended parameter cut‐off values for hard filtering) identified many (936‐5383) candidate mutations in an MA line at a time point (Table S6). The rates of cross‐line sharing of candidate mutations for time‐point pairs (1, 2), (1, 3), and (2, 3) were 0.041, 0.068, and 0.086, respectively (Figure 1a). The rates of within‐line sharing of candidate mutations for time‐point pairs (1, 2), (1, 3), and (2, 3) were 0.007, 0.010, and 0.022, respectively (Figure 1b). These results indicate that GATK has a higher false discovery rate and a substantially lower positive predictive value of mutation identification compared to BMF (Figure 3a,b). When adjusting the cut‐off values based on the distribution of the parameter values, the numbers of candidate mutations became more reasonable (Tables S3 and S6). However, the rates of candidate mutations with the adjusted cut‐off values (Figures 1 and 3a,b) remained higher for cross‐line sharing and lower for within‐line sharing than those using BMF. The results using parameter cut‐off values based on histograms of the parameter values and cut‐off values used in published studies (Figure S3) and those requiring at least six nucleotide reads to call genotypes (Figure S4) also showed a higher false discovery rate and a lower positive predictive value by GATK than BMF. Compared to the positive predictive value when requiring at least six nucleotide reads to call genotypes (Figure S4b), the corresponding value when requiring at least eight nucleotide reads to call genotypes (Figure 3b) was higher, indicating more confirmed candidate mutations when requiring at least eight nucleotide reads to call individual genotypes.

In total, there were 36,709 true single nucleotide mutations in the simulated data with moderate (mean 10× specified) depths of coverage. A small fraction (215 out of 36,706 true mutations) were at callable sites with BMF. We identified 225 candidate single nucleotide mutations using BMF. Of these, 205 were true positives and 20 were false positives. Using GATK and hard filtering with generally recommended parameter cut‐off values, we identified 212 candidate single nucleotide mutations. Of these, 184 were true positives and 28 were false positives. Integrative Genomics Viewer inspection did not find any signature of mismapping around the false‐positive candidate mutations by both BMF and GATK and the genotype quality of the MA line containing each GATK false‐positive candidate mutation was relatively high (≥43), revealing the difficulty of distinguishing sequencing errors from true mutations with moderate depths of coverage. Consistent with the results with empirical data, BMF had a lower false discovery rate (0.09 vs. 0.13; Figure 3c) than GATK. Of the 215 true single nucleotide mutations at callable sites, 10 were not identified by BMF, indicating a false‐negative rate of 0.05 by BMF. Of the 192 true single nucleotide mutations at callable sites, 8 were not identified by GATK, indicating a slightly lower false‐negative rate of 0.04 by GATK than BMF (Figure 3d). Using adjusted cut‐off values in hard filtering, none of 136 candidate mutations by GATK was false (Figure 3c). However, of the 192 true single nucleotide mutations at callable sites, 56 were not identified by GATK with adjusted parameter cut‐off values, indicating a high false‐negative rate of 0.29 (Figure 3d). With higher depths of coverage (mean 30× specified), large fractions (30,736 and 30,796 with BMF and GATK, respectively, out of 36,915) of true mutations were at callable sites. Of 30,189 candidate single nucleotide mutations identified using BMF, 15 were false positives, indicating a false‐positive rate of 0.0005 by BMF (Figure S5a). Of 29,537 candidate single nucleotide mutations identified using GATK with generally recommended parameter cut‐off values in hard filtering, 10 were false positives, indicating a false‐positive rate of 0.0003 by GATK with generally recommended parameter cut‐off values in hard filtering (Figure S5a). Of the 30,736 true single nucleotide mutations at callable sites, 562 were not identified by BMF, indicating a false‐negative rate of 0.02 by BMF (Figure S5b). Of the 30,796 true single nucleotide mutations at callable sites, 1269 were not identified by GATK with generally recommended parameter cut‐off values in hard filtering, indicating a false‐negative rate of 0.04 by GATK with generally recommended parameter cut‐off values in hard filtering (Figure S5b). Using adjusted cut‐off values in hard filtering, none of 26,698 candidate mutations by GATK was false (Figure S5a). However, of the 30,796 true single nucleotide mutations at callable sites, 4098 were not identified by GATK with adjusted parameter cut‐off values, indicating a relatively high false‐negative rate of 0.13 (Figure S5b).

### 3.6 Computing time and memory

Starting from the same BAM files of the 14 MA lines at time point one, genotype calling using GATK took 802 h and 34.75 GB of memory, whereas that using BMF took only 6.11 h and 0.07 GB of memory when analyzing the data under the same computing environment (Figure 3e,f). Thus, in terms of the computing time and memory used, BMF was 131 and 464 times more efficient than GATK, respectively.

## 4 DISCUSSION

To take maximum advantage of the high‐throughput sequencing technologies for investigating single nucleotide mutations, a systematic statistical method that enables accurate and reproducible mutation identification is essential. Time‐series MA data enable performance evaluation of methods for identifying mutations under the complexities of empirical data. Considering the characteristics of MA data generated using high‐throughput sequencing technologies, we adapted the existing BGC (Maruki & Lynch, 2017) in BMF and assessed its performance with time‐series MA and simulated data. The performance comparison of BMF and the widely used GATK indicated that BMF enables more accurate identification of single nucleotide mutations than GATK. This result was even more evident when applied to the empirical data. Compared to existing methods, BMF involves fewer parameters requiring adjustments specific to analyzed data.

### 4.1 Mutation rate estimates

Our mutation rate estimates at time points one (6.44 × 10^−9^ per site per generation per line) and two (2.22 × 10^−9^ per site per generation per line) are similar but somewhat higher than previous estimates at time points one (2.30 × 10^−9^ per site per generation per line) (Flynn et al., 2017) and two (1.61 × 10^−9^ per site per generation per line) (Bull et al., 2019), respectively. This is likely due to the more stringent filtering procedures used in previous studies than the filtering used in this study. The mean of our mutation rate estimates over the time points (4.01 × 10^−9^ per site per generation per line) agrees well with the previous estimate for *Daphnia pulex* (3.8 × 10^−9^ per site per generation per line) based on high coverage (mean 36×) sequencing data by Keith et al. (2016). Considering the high rate of unconfirmed candidate mutations and higher false discovery rate than the false‐negative rate with simulated data with moderate depths of coverage, the mutation rate estimate based on all candidate mutations is probably higher than the true mutation rate. Therefore, we provide the mean of the mutation rate estimates over time‐point pairs using sites callable at both time points (1.00 × 10^−9^ ± 5.65 × 10^−11^ per site per generation per line, where the standard error of the mean is also shown) as our updated mutation rate estimate for *D. pulex*.

### 4.2 Unconfirmed candidate mutations

One of the most surprising results in this study is the relatively high rate of unconfirmed candidate mutations at later time points with BMF and particularly with GATK. The rates of within‐line sharing of candidate mutations were generally low and GATK had substantially lower rates than BMF (Figure 1b). The unconfirmed candidate mutations are mainly due to the absence of the mutant nucleotide read at later time points (Table S5). Alternative hypotheses for the missing mutant nucleotide read at later time points (Table 3) include H1) removal of the mutant nucleotide by the filtering procedure; H2) incorrect identity of some MA lines; H3) segregating polymorphisms; H4) mismapping of sequence reads around the sites with unconfirmed candidate mutations; H5) errors in sequencing and/or library preparation at the time point when the candidate mutation was identified; H6) back mutations; H7) sequencing of only the chromosome with the ancestral allele; H8) identification of somatic mutations; H9) gene conversion or hemizygous deletions.

**TABLE 3:** Alternative hypotheses for the unconfirmed candidate mutations.

Hypothesis	Main cause?	Reason
1) Removal of the mutant nucleotide by the filtering	No	Mutant nucleotide not found without filtering
2) Incorrect identity of some MA lines	No	Identity of all MA lines confirmed by unique candidate mutations introduced at time point one
3) Segregating polymorphisms	No	Mutation rates are generally low and the isolates in each MA line are genetically identical most of the time
4) Mismapping of sequence reads	Likely	Some (7%) unconfirmed candidate mutations show signatures of mismapping (Tables S3 and S5). One read of the mutant nucleotide is sometimes (38%) found in another MA line at a later time point (Table S5)
5) Errors in sequencing and/or library preparation	Likely	One read of the mutant nucleotide is sometimes (38%) found in another MA line at a later time point (Table S5)
6) Back mutation	No	Mutation rates are generally too low for mutations to occur twice at a particular site
7) Sequencing of only the chromosome with the ancestral allele	Unknown	Only the chromosome containing the ancestral allele can be sequenced with moderate depths of coverage
8) Identification of somatic mutations	No	Nucleotide reads from somatic mutations should deviate from the binomial expectation
9) Gene conversion or hemizygous deletion	No	None of the candidate mutations was in a homozygous genotype call

To examine whether the mutant nucleotide read was removed by the filtering procedure (H1), we examined the nucleotide read counts just after mapping. The mutant nucleotide read was missing at later time points at most sites without filtering and therefore the filtering procedure is not the main cause of the missing mutant nucleotide read.

To rule out the possibility of incorrect identity of MA lines (H2), we examined the identity of the MA lines at time points two and three using unique candidate mutations characterizing each line identified at time point one. The identity of each MA line was confirmed by at least one unique candidate mutation supported by at least two mutant nucleotide reads at time points two and three, indicating that the incorrect identity is unlikely to be the cause of the missing mutant nucleotide read at later time points.

Polymorphisms segregating within MA lines (H3) could result in identification of unconfirmed candidate mutations. Our MA lines derived from an obligatory asexual isolate and only one neonate was transferred in each generation. More than one neonate may have been occasionally transferred but segregating variants are unlikely to be the main cause underlying many unconfirmed candidate mutations because mutation rates are generally low and the isolates within each of our MA lines are typically genetically identical.

To examine mismapping of sequence reads (H4), we compared mean depths of coverage between sites with confirmed and unconfirmed candidate mutations using the two‐tailed two‐sample *t*‐test. The mean total coverage at sites with confirmed candidate mutations (281 and 237 at time points two and three, respectively) was not significantly different from that at sites with unconfirmed candidate mutations (274 and 229 at time points two and three, respectively) (*p* > .05). However, IGV inspection of the unconfirmed candidate mutations found signatures of mismapping (nearby variants in complete association on the same sequence read that are also found in other MA lines; Keightley et al., 2014) (Table S3) in some sequence reads, indicating that some of the unconfirmed candidate mutations may be due to mismapping of sequence reads. If additional mutations had occurred around the unconfirmed candidate mutation, sequence reads containing the unconfirmed candidate mutation may fail to map to the reference sequence due to reference mapping bias (Günther & Nettelblad, 2019; Ros‐Freixedes et al., 2018). Under this scenario, depths of coverage should be lower at sites with unconfirmed candidate mutations. However, the mean total coverage (274 and 229 at time points two and three, respectively) is somewhat higher or similar compared to that at analyzed sites (254 and 225 at time points two and three, respectively).

To examine errors in sequencing and/or library preparation (H5), we calculated the mean error‐rate estimate over the analyzed sites at each time point. The mean error‐rate estimates at time points one, two, and three were 3.4 × 10^−4^, 2.9 × 10^−4^, and 3.1 × 10^−4^, respectively. These results are in line with the sequencing error rates with Illumina platforms (Stoler & Nekrutenko, 2021). In contrast to the similar mean error‐rate estimates among the time points over the analyzed sites, the mean error‐rate estimate over sites with unconfirmed candidate mutations was significantly higher at time points two and three (3.15 × 10^−3^ and 2.63 × 10^−3^, respectively) than at time point one (1.8 × 10^−4^). Close examination of the input nucleotide read counts (Table S5) revealed that this is because one read of the nucleotide supporting the unconfirmed candidate mutation identified at time point one tends to be found in another MA line at a later time point, indicating that errors in sequencing or read mapping may be the cause of some unconfirmed candidate mutations. Because the same nucleotide read as the mutant nucleotide read was found in another MA line, this observation is more likely to be due to errors in read mapping than sequencing.

Given that the mutation rate is generally low, back mutation (H6) is unlikely to be the main cause of the missing nucleotide read at later time points. Assuming that the mutation rate is 1.00 × 10^−9^ per site per generation per line and mutations to the three different nucleotides are equally likely, the probability that back mutation converts the mutant nucleotide to the ancestral nucleotide at a given site is 3.34 × 10^−10^ in each generation.

Sequencing of only the chromosome with the ancestral allele (H7) may be the cause at some of the sites with moderate coverage but is unlikely to be the main cause given that many of the sites with the missing mutant nucleotide read at later time points had relatively high coverage in the MA line where the candidate mutation was identified at an earlier time point.

Because somatic mutations (H8) are limited to mutated cells, the nucleotide read counts from somatic mutants are therefore expected to significantly deviate from the binomial expectation. Thus, somatic mutation is unlikely to be the main cause.

As the MA line with the missing mutant nucleotide read at later time points has heterozygous sites within 900 bp of the site with the missing candidate mutation for more than a half of the cases (Figure S6), gene conversion or hemizygous deletions (H9) is unlikely to be the main cause. Moreover, given that depths of coverage in the MA line are normal around sites with the missing candidate mutations, hemizygous deletion is unlikely to explain the observed pattern. All candidate mutations were in heterozygous genotype calls, which makes gene conversion or hemizygous deletions unlikely to be the main cause, as some candidate mutations are expected to be found in homozygous genotype calls if mutations followed by gene conversion or hemizygous deletions occur.

Considering all the hypotheses, mismapping of sequence reads (H4) appears the most likely cause of the unconfirmed candidate mutations. As depths of coverage were normal at sites with unconfirmed candidate mutations and only some (7%) of the candidate mutations were found in sequence reads showing signatures of mismapping (Tables S3 and S5), this study highlights the difficulty of identifying false positives resulting from mismapping. This finding is also consistent with the low rate of unconfirmed candidate mutations first identified at time point two (0.16) given the availability of BMF genotype calls compared to the corresponding rate for candidate mutations first identified at time point one. It is important to note the longer sequence reads (150 bp) at time points two and three than at time point one (100 bp) (Table 1) as the length of the sequence reads is known to influence the quality of the mapping.

In empirical data, large‐scale duplications present in MA lines but not in the reference sequence exist (Chain et al., 2019), causing more mismapping and lower positive predictive values in empirical than simulation results. Given the overall high rate of unconfirmed candidate mutations, the false discovery rate of mutation identification with the empirical data is probably high. This is because of the fact that nucleotide reads resulting from mismapping or sequencing errors resemble true mutations with moderate depths of coverage. Furthermore, in our independently evolving MA lines derived from a single asexual isolate, each de novo mutation is expected to be found in a heterozygous genotype, which is much more difficult to correctly call than a homozygous one (Maruki & Lynch, 2017). Our time‐series MA data enabled genome‐wide validation of candidate mutations and rigorous mutation rate estimation based on candidate mutations confirmed by subsequent sequencing.

## 5 CONCLUSIONS

Our finding is consistent with that by Lefouili and Nam (2022), who found that GATK had many false positives resulting from mismapping of sequence reads. Although adjusting the cut‐off values based on the distribution of the parameters reduced the number of candidate mutations, the cross‐line and within‐line sharing of candidate mutations showed only small changes, indicating that the adjustment does not substantially improve the mutation identification using GATK in terms of the false discovery rate and positive predictive value. Furthermore, how exactly GATK users should adjust the parameter cut‐off values is not clear, making it hard to compare results. Other existing methods such as muver (Burkholder et al., 2018) and accuMUlate (Winter et al., 2018) also involve many parameters, which can be difficult to specify, and are not easy to use. Since BMF does not require such adjustments, consistency of results and comparability across studies are expected to improve.

BMF enables more accurate identification of single nucleotide mutations from high‐throughput sequencing data with moderate depths of coverage than the currently widely used GATK. This is important because despite the decrease in sequencing cost, coverage remains limited in many organisms, particularly those with large genome size. Furthermore, recent studies are acknowledging the importance of including multiple individuals from multiple populations to efficiently study mutations in a species (Dumont, 2019; Gou et al., 2019; Harris, 2015; Ness et al., 2015). Thus, coverage may also remain limited in studies with such design. As coverage increases through development of high‐throughput sequencing technologies, the performance of BMF will also improve (Figure S5). Since the magnitude of high‐throughput sequencing data is rapidly increasing, the efficiency of BMF matters.

## AUTHOR CONTRIBUTIONS

**Takahiro Maruki:** Conceptualization (lead); formal analysis (lead); investigation (lead); methodology (lead); software (lead); writing – original draft (lead); writing – review and editing (equal). **April Ozere:** Formal analysis (supporting); investigation (supporting); writing – original draft (supporting); writing – review and editing (supporting). **Jack Freeman:** Investigation (supporting); writing – original draft (supporting); writing – review and editing (supporting). **Melania E. Cristescu:** Conceptualization (equal); funding acquisition (lead); project administration (lead); supervision (lead); writing – review and editing (lead).

## FUNDING INFORMATION

This work was supported by a Natural Sciences and Engineering Council of Canada (NSERC) CREATE training program on Aquatic Ecosystem Health (397997‐2011), and NSERC Discovery Grant (04331‐2017) and Canada Research Chair (23710) to MEC.

## CONFLICT OF INTEREST STATEMENT

The authors have no conflict of interest to declare.

## BENEFIT‐SHARING STATEMENT

This research benefits the community by freely sharing our data and computer programs through web sites described above.

## Supporting information


Figure S1

Figure S2

Figure S3

Figure S4

Figure S5

Figure S6

Table S1

Table S2

Table S3

Table S4

Table S5

Table S6

Text S1

Text S2

Text S3

Text S4

## Data Availability

FASTQ files of whole‐genome sequencing data of the 14 MA lines at time point one are available at the NCBI SRA (BioProject ID: PRJNA341529). FASTQ files of whole‐genome sequencing data of the 14 MA lines at time points two and three were generated in this study and are available at the NCBI SRA (BioProject ID: PRJNA847774). BMF is available through GitHub (https://github.com/Takahiro‐Maruki/BMF).

## References

[ece370339-bib-0001] Adey, A. , Morrison, H. G. , Asan, X. , Xun, J. O. , Kitzman, J. O. , Turner, E. H. , Stackhouse, B. , MacKenzie, A. P. , Caruccio, N. C. , Zhang, X. , & Shendure, J. (2010). Rapid, low‐input, low‐bias construction of shotgun fragment libraries by high‐density in vitro transposition. Genome Biology, 11, R119. 21143862 10.1186/gb-2010-11-12-r119 PMC3046479

[ece370339-bib-0002] Barnard‐Kubow, K. B. , Becker, D. , Murray, C. S. , Porter, R. , Gutierrez, G. , Erickson, P. , Nunez, J. C. B. , Voss, E. , Suryamohan, K. , Ratan, A. , Beckerman, A. , & Bergland, A. O. (2022). Genetic variation in reproductive investment across an ephemerality gradient in *Daphnia pulex* . Molecular Biology and Evolution, 39, msac121. 35642301 10.1093/molbev/msac121 PMC9198359

[ece370339-bib-0003] Baym, M. , Kryazhimskiy, S. , Lieberman, T. D. , Chung, H. , Desai, M. M. , & Kishony, R. (2015). Inexpensive multiplexed library preparation for megabase‐sized genomes. PLoS One, 10, e0128036. 26000737 10.1371/journal.pone.0128036 PMC4441430

[ece370339-bib-0004] Behringer, M. G. , & Hall, D. W. (2016). Genome‐wide estimates of mutation rates and spectrum in *Schizosaccharomyces pombe* indicate CpG sites are highly mutagenic despite the absence of DNA methylation. G3 (Bethesda), 6, 149–160. 10.1534/g3.115.022129 PMC4704713 26564949

[ece370339-bib-0005] Bergeron, L. A. , Besenbacher, S. , Turner, T. , Versoza, C. J. , Wang, R. J. , Price, A. L. , Armstrong, E. , Riera, M. , Carlson, J. , Chen, H. Y. , Hahn, M. W. , Harris, K. , Kleppe, A. S. , López‐Nandam, E. H. , Moorjani, P. , Pfeifer, S. P. , Tiley, G. P. , Yoder, A. D. , Zhang, G. , & Schierup, M. H. (2022). The Mutationathon highlights the importance of reaching standardization in estimates of pedigree‐based germline mutation rates. eLife, 11, e73577. 35018888 10.7554/eLife.73577 PMC8830884

[ece370339-bib-0006] Bolger, A. M. , Lohse, M. , & Usadel, B. (2014). Trimmomatic: A flexible trimmer for illumina sequence data. Bioinformatics, 30, 2114–2120. 24695404 10.1093/bioinformatics/btu170 PMC4103590

[ece370339-bib-0007] Bull, J. K. , Flynn, J. M. , Chain, F. J. J. , & Cristescu, M. E. (2019). Fitness and genomic consequences of chronic exposure to low levels of copper and nickel in *Daphnia pulex* mutation accumulation lines. G3 (Bethesda), 9, 61–71. 30389796 10.1534/g3.118.200797 PMC6325897

[ece370339-bib-0008] Burda, K. , & Konczal, M. (2023). Validation of machine learning approach for direct mutation rate estimation. Molecular Ecology Resources, 23, 1757–1771. 37486035 10.1111/1755-0998.13841

[ece370339-bib-0009] Burkholder, A. B. , Lujan, S. A. , Lavender, C. A. , Grimm, S. A. , Kunkel, T. A. , & Fargo, D. C. (2018). Muver, a computational framework for accurately calling accumulated mutations. BMC Genomics, 19, 345. 29743009 10.1186/s12864-018-4753-3 PMC5944071

[ece370339-bib-0010] Chain, F. J. J. , Flynn, J. M. , Bull, J. K. , & Cristescu, M. E. (2019). Accelerated rates of large‐scale mutations in the presence of copper and nickel. Genome Research, 29, 64–73. 30487211 10.1101/gr.234724.118 PMC6314161

[ece370339-bib-0011] Colbourne, J. K. , Pfrender, M. E. , Gilbert, D. , Thomas, W. K. , Tucker, A. , Oakley, T. H. , Tokishita, S. , Aerts, A. , Arnold, G. J. , Basu, M. K. , Bauer, D. J. , Cáceres, C. E. , Carmel, L. , Casola, C. , Choi, J. H. , Detter, J. C. , Dong, Q. , Dusheyko, S. , Eads, B. D. , … Boore, J. L. (2011). The ecoresponsive genome of *Daphnia pulex* . Science, 331, 555–561. 21292972 10.1126/science.1197761 PMC3529199

[ece370339-bib-0012] Crease, T. J. (1999). The complete sequence of the mitochondrial genome of *Daphnia pulex* (Cladocera: Crustacea). Gene, 233, 89–99. 10375625 10.1016/s0378-1119(99)00151-1

[ece370339-bib-0013] DePristo, M. A. , Banks, E. , Poplin, R. , Garimella, K. V. , Maguire, J. R. , Hartl, C. , Philippakis, A. A. , del Angel, G. , Rivas, M. A. , Hanna, M. , McKenna, A. , Fennell, T. J. , Kernytsky, A. M. , Sivachenko, A. Y. , Cibulskis, K. , Gabriel, S. B. , Altshuler, D. , & Daly, M. J. (2011). A framework for variation discovery and genotyping using next‐generation DNA sequencing data. Nature Genetics, 43, 491–498. 21478889 10.1038/ng.806 PMC3083463

[ece370339-bib-0014] Doyle, J. J. , & Doyle, J. L. (1987). A rapid DNA isolation procedure for small quantities of fresh leaf tissue. Phytochemical Bulletin, 19, 11–15.

[ece370339-bib-0015] Dumont, B. L. (2019). Significant strain variation in the mutation spectra of inbred laboratory mice. Molecular Biology and Evolution, 36, 865–874. 30753674 10.1093/molbev/msz026 PMC6501876

[ece370339-bib-0016] Fields, P. D. , Reisser, C. , Dukic, M. , Haag, C. R. , & Ebert, D. (2015). Genes mirror geography in *Daphnia magna* . Molecular Ecology, 24, 4521–4536. 26190313 10.1111/mec.13324

[ece370339-bib-0017] Flynn, J. M. , Chain, F. J. , Schoen, D. J. , & Cristescu, M. E. (2017). Spontaneous mutation accumulation in *Daphnia pulex* in selection‐free vs. competitive environments. Molecular Biology and Evolution, 34, 160–173. 27777284 10.1093/molbev/msw234

[ece370339-bib-0018] Gou, L. , Bloom, J. S. , & Kruglyak, L. (2019). The genetic basis of mutation rate variation in yeast. Genetics, 211, 731–740. 30504363 10.1534/genetics.118.301609 PMC6366923

[ece370339-bib-0019] Günther, T. , & Nettelblad, C. (2019). The presence and impact of reference bias on population genomic studies of prehistoric human populations. PLoS Genetics, 15, e1008302. 31348818 10.1371/journal.pgen.1008302 PMC6685638

[ece370339-bib-0020] Halligan, D. L. , & Keightley, P. D. (2009). Spontaneous mutation accumulation studies in evolutionary genetics. Annual Review of Ecology, Evolution, and Systematics, 40, 151–172.

[ece370339-bib-0021] Harris, K. (2015). Evidence for recent, population‐specific evolution of the human mutation rate. Proceedings of the National Academy of Sciences of the United States of America, 112, 3439–3444. 25733855 10.1073/pnas.1418652112 PMC4371947

[ece370339-bib-0022] Hebert, P. D. N. , & Crease, T. J. (1980). Clonal coexistence in *Daphnia pulex* (Leydig): Another planktonic paradox. Science, 207, 1363–1365.

[ece370339-bib-0023] Hebert, P. D. N. , & Crease, T. J. (1983). Clonal diversity in populations of *Daphnia pulex* reproducing by obligate parthenogenesis. Heredity, 51, 353–369.

[ece370339-bib-0024] Ho, E. K. H. , Macrae, F. , Latta, L. C. , McIlroy, P. , Ebert, D. , Fields, P. D. , Benner, M. J. , & Schaack, S. (2020). High and highly variable spontaneous mutation rates in *Daphnia* . Molecular Biology and Evolution, 37, 3258–3266. 32520985 10.1093/molbev/msaa142 PMC7820357

[ece370339-bib-0025] Innes, D. J. , & Hebert, P. D. N. (1988). The origin and genetic basis of obligate parthenogenesis in *Daphnia pulex* . Evolution, 42, 1024–1035. 28581165 10.1111/j.1558-5646.1988.tb02521.x

[ece370339-bib-0026] Katju, V. , & Bergthorsson, U. (2019). Old trade, new tricks: Insights into the spontaneous mutation process from the partnering of classical mutation accumulation experiments with high‐throughput genomic approaches. Genome Biology and Evolution, 11, 136–165. 30476040 10.1093/gbe/evy252 PMC6330053

[ece370339-bib-0027] Keightley, P. D. , Ness, R. W. , Halligan, D. L. , & Haddrill, P. R. (2014). Estimation of the spontaneous mutation rate per nucleotide site in a *Drosophila melanogaster* full‐sib family. Genetics, 196, 313–320. 24214343 10.1534/genetics.113.158758 PMC3872194

[ece370339-bib-0028] Keightley, P. D. , Trivedi, U. , Thomson, M. , Oliver, F. , Kumar, S. , & Blaxter, M. L. (2009). Analysis of the genome sequences of three *Drosophila melanogaster* spontaneous mutation accumulation lines. Genome Research, 19, 1195–1201. 19439516 10.1101/gr.091231.109 PMC2704435

[ece370339-bib-0029] Keith, N. , Jackson, C. E. , Glaholt, S. P. , Young, K. , Lynch, M. , & Shaw, J. R. (2021). Genome‐wide analysis of cadmium‐induced, germline mutations in a long‐term *Daphnia pulex* mutation‐accumulation experiment. Environmental Health Perspectives, 129, 107003. 34623885 10.1289/EHP8932 PMC8500294

[ece370339-bib-0030] Keith, N. , Tucker, A. E. , Jackson, C. E. , Sung, W. , Lucas Lledo, J. I. , Schrider, D. R. , Schaack, S. , Dudycha, J. L. , Ackerman, M. , Younge, A. J. , Shaw, J. R. , & Lynch, M. (2016). High mutational rates of large‐scale duplication and deletion in *Daphnia pulex* . Genome Research, 26, 60–69. 26518480 10.1101/gr.191338.115 PMC4691751

[ece370339-bib-0031] Kondrashov, A. S. (1988). Deleterious mutations and the evolution of sexual reproduction. Nature, 336, 435–440. 3057385 10.1038/336435a0

[ece370339-bib-0032] Kondrashov, F. A. , & Kondrashov, A. S. (2010). Measurements of spontaneous rates of mutations in the recent past and the near future. Philosophical Transactions of the Royal Society of London. Series B, Biological Sciences, 365, 1169–1176. 20308091 10.1098/rstb.2009.0286 PMC2871817

[ece370339-bib-0033] Konrad, A. , Brady, M. J. , Bergthorsson, U. , & Katju, V. (2019). Mutational landscape of spontaneous base substitutions and small indels in experimental *Caenorhabditis elegans* populations of differing size. Genetics, 212, 837–854. 31110155 10.1534/genetics.119.302054 PMC6614903

[ece370339-bib-0034] Lefouili, M. , & Nam, K. (2022). The evaluation of Bcftools mpileup and GATK HaplotypeCaller for variant calling in non‐human species. Scientific Reports, 12, 11331. 35790846 10.1038/s41598-022-15563-2 PMC9256665

[ece370339-bib-0035] Li, H. , Handsaker, B. , Wysoker, A. , Fennell, T. , Ruan, J. , Homer, N. , Marth, G. , Abecasis, G. , Durbin, R. , & 1000 Genome Project Data Processing Subgroup . (2009). The sequence alignment/map format and SAMtools. Bioinformatics, 25, 2078–2079. 19505943 10.1093/bioinformatics/btp352 PMC2723002

[ece370339-bib-0036] Loewe, L. , & Hill, W. G. (2010). The population genetics of mutations: Good, bad and indifferent. Philosophical Transactions of the Royal Society of London. Series B, Biological Sciences, 365, 1153–1167. 20308090 10.1098/rstb.2009.0317 PMC2871823

[ece370339-bib-0037] Long, H. , Winter, D. J. , Chang, A. Y. , Sung, W. , Wu, S. H. , Balboa, M. , Azevedo, R. B. R. , Cartwright, R. A. , Lynch, M. , & Zufall, R. A. (2016). Low base‐substitution mutation rate in the germline genome of the ciliate *Tetrahymena thermophil* . Genome Biology and Evolution, 8, 3629–3639. 27635054 10.1093/gbe/evw223 PMC5585995

[ece370339-bib-0038] Lynch, M. , Ackerman, M. S. , Gout, J. F. , Long, H. , Sung, W. , Thomas, W. K. , & Foster, P. L. (2016). Genetic drift, selection and the evolution of the mutation rate. Nature Reviews. Genetics, 17, 704–714. 10.1038/nrg.2016.104 27739533

[ece370339-bib-0039] Maruki, T. , & Lynch, M. (2015). Genotype‐frequency estimation from high‐throughput sequencing aata. Genetics, 201, 473–486. 26224735 10.1534/genetics.115.179077 PMC4596663

[ece370339-bib-0040] Maruki, T. , & Lynch, M. (2017). Genotype calling from population‐genomic sequencing data. G3 (Bethesda), 7, 1393–1404. 28108551 10.1534/g3.117.039008 PMC5427492

[ece370339-bib-0041] McDonald, J. H. (2014). Handbook of biological statistics (3rd ed.). Sparky House Publishing.

[ece370339-bib-0042] McKenna, A. , Hanna, M. , Banks, E. , Sivachenko, A. , Cibulskis, K. , Kernytsky, A. , Garimella, K. , Altshuler, D. , Gabriel, S. , Daly, M. , & DePristo, M. A. (2010). The Genome Analysis Toolkit: A MapReduce framework for analyzing next‐generation DNA sequencing data. Genome Research, 20, 1297–1303. 20644199 10.1101/gr.107524.110 PMC2928508

[ece370339-bib-0043] Ness, R. W. , Morgan, A. D. , Vasanthakrishnan, R. B. , Colegrave, N. , & Keightley, P. D. (2015). Extensive de novo mutation rate variation between individuals and across the genome of *Chlamydomonas reinhardtii* . Genome Research, 25, 1739–1749. 26260971 10.1101/gr.191494.115 PMC4617969

[ece370339-bib-0044] Ros‐Freixedes, R. , Battagin, M. , Johnsson, M. , Gorjanc, G. , Mileham, A. J. , Rounsley, S. D. , & Hickey, J. M. (2018). Impact of index hopping and bias towards the reference allele on accuracy of genotype calls from low‐coverage sequencing. Genetics, Selection, Evolution, 50, 64. 10.1186/s12711-018-0436-4 PMC6293637 30545283

[ece370339-bib-0045] Stephens, Z. D. , Hudson, M. E. , Mainzer, L. S. , Taschuk, M. , Weber, M. R. , & Iyer, R. K. (2016). Simulating next‐generation sequencing datasets from empirical mutation and sequencing models. PLoS One, 11, e0167047. 27893777 10.1371/journal.pone.0167047 PMC5125660

[ece370339-bib-0046] Stoler, N. , & Nekrutenko, A. (2021). Sequencing error profiles of illumina sequencing instruments. NAR Genomics and Bioinformatics, 3, lqab019. 33817639 10.1093/nargab/lqab019 PMC8002175

[ece370339-bib-0047] Thorvaldsdottir, H. , Robinson, J. T. , & Mesirov, J. P. (2013). Integrative genomics viewer (IGV): High‐performance genomics data visualization and exploration. Briefings in Bioinformatics, 14, 178–192. 22517427 10.1093/bib/bbs017 PMC3603213

[ece370339-bib-0048] Van der Auwera, G. A. , Carneiro, M. O. , Hartl, C. , Poplin, R. , Del Angel, G. , Levy‐Moonshine, A. , Jordan, T. , Shakir, K. , Roazen, D. , Thibault, J. , Banks, E. , Garimella, K. V. , Altshuler, D. , Gabriel, S. , & DePristo, M. A. (2013). From FastQ data to high confidence variant calls: The genome analysis toolkit best practices pipeline. Current Protocols in Bioinformatics, 11, 11.10.11–11.10.33. 10.1002/0471250953.bi1110s43 PMC4243306 25431634

[ece370339-bib-0049] Winter, D. J. , Wu, S. H. , Howell, A. A. , Azevedo, R. B. R. , Zufall, R. A. , & Cartwright, R. A. (2018). accuMUlate: A mutation caller designed for mutation accumulation experiments. Bioinformatics, 34, 2659–2660. 29566129 10.1093/bioinformatics/bty165 PMC6061804

[ece370339-bib-0050] Xu, S. , Schaack, S. , Seyfert, A. , Choi, E. , Lynch, M. , & Cristescu, M. E. (2012). High mutation rates in the mitochondrial genomes of *Daphnia pulex* . Molecular Biology and Evolution, 29, 763–769. 21998274 10.1093/molbev/msr243 PMC3350313

[ece370339-bib-0051] Ye, Z. , Jiang, X. , Pfrender, M. E. , & Lynch, M. (2021). Genome‐wide allele‐specific expression in obligately asexual *Daphnia pulex* and the implications for the genetic basis of asexuality. Genome Biology and Evolution, 13, evab243. 34726699 10.1093/gbe/evab243 PMC8598174

[ece370339-bib-0052] Ye, Z. , Xu, S. , Spitze, K. , Asselman, J. , Jiang, X. , Ackerman, M. S. , Lopez, J. , Harker, B. , Raborn, R. T. , Thomas, W. K. , Ramsdell, J. , Pfrender, M. E. , & Lynch, M. (2017). A new reference genome assembly for the microcrustacean *Daphnia pulex* . G3 (Bethesda), 7, 1405–1416. 28235826 10.1534/g3.116.038638 PMC5427498

