# Supplementary material for: What can we infer about mutation calling by using time‐series mutation accumulation data and a Bayesian Mutation Finder?
Source: Ecol Evol. 2024 Nov 10;14(11):e70339. doi: 10.1002/ece3.70339 (PMC11550904; doi:10.1002/ece3.70339)
Supplement: Supplementary file 2 — Figure S2 [file ECE3-14-e70339-s005.docx]

Supporting Information for:

What can we infer about mutation calling by using time-series mutation accumulation data and a Bayesian Mutation Finder?

Takahiro Maruki, April Ozere, Jack Freeman, and Melania E. Cristescu

**Figure S2** Histograms of the hard-filtering parameters in the SNP VCF files of GATK genotype calls. Frequency distribution of QD, QUAL, SOR, FS, MQ, MQRankSum, and ReadPosRankSum in the single nucleotide polymorphism (SNP) VCF file of GATK genotype calls before hard filtering at each of the three time points is shown.
