# Supplementary material for: What can we infer about mutation calling by using time‐series mutation accumulation data and a Bayesian Mutation Finder?
Source: Ecol Evol. 2024 Nov 10;14(11):e70339. doi: 10.1002/ece3.70339 (PMC11550904; doi:10.1002/ece3.70339)
Supplement: Supplementary file 3 — Figure S3 [file ECE3-14-e70339-s007.docx]

Supporting Information for:

What can we infer about mutation calling by using time-series mutation accumulation data and a Bayesian Mutation Finder?

Takahiro Maruki, April Ozere, Jack Freeman, and Melania E. Cristescu

**Figure S3** Performance comparison of BMF and GATK with another set of adjusted parameter cut-off values in hard filtering. The A) false discovery rate and B) positive predictive value with empirical time-series MA data calculated with pooled data across time-point pairs are shown. GATK results are shown with generally recommended parameter cut-off values and adjusted cut-off values in hard filtering chosen based on histograms of the parameters and cut-off values used in published studies.
