# Supplementary material for: What can we infer about mutation calling by using time‐series mutation accumulation data and a Bayesian Mutation Finder?
Source: Ecol Evol. 2024 Nov 10;14(11):e70339. doi: 10.1002/ece3.70339 (PMC11550904; doi:10.1002/ece3.70339)
Supplement: Supplementary file 4 — Figure S4 [file ECE3-14-e70339-s015.docx]

Supporting Information for:

What can we infer about mutation calling by using time-series mutation accumulation data and a Bayesian Mutation Finder?

Takahiro Maruki, April Ozere, Jack Freeman, and Melania E. Cristescu

**Figure S4** Performance comparison of BMF and GATK requiring at least six nucleotide reads to call genotypes. The A) false discovery rate and B) positive predictive value with empirical time-series MA data calculated with pooled data across time-point pairs are shown. GATK results are shown with generally recommended parameter cut-off values and adjusted cut-off values in hard filtering.
