# Supplementary material for: What can we infer about mutation calling by using time‐series mutation accumulation data and a Bayesian Mutation Finder?
Source: Ecol Evol. 2024 Nov 10;14(11):e70339. doi: 10.1002/ece3.70339 (PMC11550904; doi:10.1002/ece3.70339)
Supplement: Supplementary file 6 — Figure S6 [file ECE3-14-e70339-s004.docx]

Supporting Information for:

What can we infer about mutation calling by using time-series mutation accumulation data and a Bayesian Mutation Finder?

Takahiro Maruki, April Ozere, Jack Freeman, and Melania E. Cristescu

**Figure S6** Distribution of the distance between unconfirmed candidate mutations and the nearest heterozygous sites. Bin width = 100 bp. Median = 855 bp.
