# Supplementary material for: What can we infer about mutation calling by using time‐series mutation accumulation data and a Bayesian Mutation Finder?
Source: Ecol Evol. 2024 Nov 10;14(11):e70339. doi: 10.1002/ece3.70339 (PMC11550904; doi:10.1002/ece3.70339)
Supplement: Supplementary file 13 — Text S1 [file ECE3-14-e70339-s003.docx]

Supporting Information for:

What can we infer about mutation calling by using time-series mutation accumulation data and a Bayesian Mutation Finder?

Takahiro Maruki, April Ozere, Jack Freeman, and Melania E. Cristescu

**Text S1** Data processing protocol.

1. Concatenate FASTA files of PA42.4.2 and *Daphnia pulex* mtDNA sequence.

cat PA42.4.2.fasta mtDNA_pulex.fasta > PA42.4.2_mtDNA.fasta

2. Make BWA and Samtools index files and a Picard dictionary file of the reference file.

bwa index PA42.4.2_mtDNA.fasta

samtools faidx PA42.4.2_mtDNA.fasta

gatk CreateSequenceDictionary -R PA42.4.2_mtDNA.fasta -O PA42.4.2_mtDNA.dict

3. After finding adaptor types (e.g., Nextera Transposase Sequence) using FastQC, trim adapter sequences from the FASTQ files using Trimmomatic.

java -jar trimmomatic-0.39.jar PE -threads 16 C001-88_R1.fastq C001-88_R2.fastq C001-88_R1-paired.fastq C001-88_R1-unpaired.fastq C001-88_R2-paired.fastq C001-88_R2-unpaired.fastq HEADCROP:3 ILLUMINACLIP: NexteraPE-PE.fa:2:30:10:2 SLIDINGWINDOW:4:15 MINLEN:30

4. Map sequence reads to the reference sequence using BWA.

bwa mem -t 16 -M PA42.4.2_mtDNA.fasta C001-88_R1-paired.fastq C001-88_R2-paired.fastq > C001-88_PA42.4.2_mtDNA-paired.sam

bwa mem -t 16 -M PA42.4.2_mtDNA.fasta C001-88_R1-unpaired.fastq > C001-88_PA42.4.2_mtDNA_R1-unpaired.sam

bwa mem -t 16 -M PA42.4.2_mtDNA.fasta C001-88_R2-unpaired.fastq > C001-88_PA42.4.2_mtDNA_R2-unpaired.sam

5. Combine the SAM files using GATK.

gatk MergeSamFiles -I C001-88_PA42.4.2_mtDNA-paired.sam -I C001-88_PA42.4.2_mtDNA_R1-unpaired.sam -I C001-88_PA42.4.2_mtDNA_R2-unpaired.sam -O C001-88_PA42.4.2_mtDNA.sam

6. Convert the SAM file to the BAM file using Samtools.

samtools view -bS C001-88_PA42.4.2_mtDNA.sam > C001-88_PA42.4.2_mtDNA.bam

7. Sort the BAM file using GATK.

gatk SortSam -I C001-88_PA42.4.2_mtDNA.bam -O Sorted_C001-88_PA42.4.2_mtDNA.bam -SORT_ORDER coordinate

8. Filter the BAM file using Samtools.

samtools view -q 20 -f 3 -F 3844 -b Sorted_C001-88_PA42.4.2_mtDNA.bam > Filtered_Sorted_C001-88_PA42.4.2_mtDNA.bam

9. Add read groups to the filtered BAM file using GATK.

gatk AddOrReplaceReadGroups -I Filtered_Sorted_C001-88_PA42.4.2_mtDNA.bam -O RG_Filtered_Sorted_C001-88_PA42.4.2_mtDNA.bam -RGID Daphnia -RGLB bar -RGPL illumina -RGSM C001-88 -RGPU 6

10. Mark duplicate reads using GATK.

gatk MarkDuplicates -I RG_Filtered_Sorted_C001-88_PA42.4.2_mtDNA.bam -O dedup_RG_Filtered_Sorted_C001-88_PA42.4.2_mtDNA.bam -M C001-88_PA42.4.2_mtDNA_metrics.txt

11. Index the BAM file using GATK.

gatk BuildBamIndex -I dedup_RG_Filtered_Sorted_C001-88_PA42.4.2_mtDNA.bam

12. Clip overlapping read pairs using bamUtil.

bam clipOverlap --in dedup_RG_Filtered_Sorted_C001-88_PA42.4.2_mtDNA.bam --out clipped_dedup_RG_Filtered_Sorted_C001-88_PA42.4.2_mtDNA.bam

13. Index the clipped BAM file using Samtools, excluding mtDNA from the BAM file.

samtools index clipped_dedup_RG_Filtered_Sorted_C001-88_PA42.4.2_mtDNA.bam

samtools idxstats clipped_dedup_RG_Filtered_Sorted_C001-88_PA42.4.2_mtDNA.bam | cut -f 1 | grep -v mitochondrial | xargs samtools view -b clipped_dedup_RG_Filtered_Sorted_C001-88_PA42.4.2_mtDNA.bam > Nuc_clipped_dedup_RG_Filtered_Sorted_C001-88_PA42.4.2_mtDNA.bam

samtools index Nuc_clipped_dedup_RG_Filtered_Sorted_C001-88_PA42.4.2_mtDNA.bam
