# Supplementary material for: What can we infer about mutation calling by using time‐series mutation accumulation data and a Bayesian Mutation Finder?
Source: Ecol Evol. 2024 Nov 10;14(11):e70339. doi: 10.1002/ece3.70339 (PMC11550904; doi:10.1002/ece3.70339)
Supplement: Supplementary file 14 — Text S2 [file ECE3-14-e70339-s002.docx]

Supporting Information for:

What can we infer about mutation calling by using time-series mutation accumulation data and a Bayesian Mutation Finder?

Takahiro Maruki, April Ozere, Jack Freeman, and Melania E. Cristescu

**Text S2** BMF protocol.

1. Make an mpileup file from the processed BAM files using the mpileup function of Samtools (Li *et al*. 2009).

a. Make a list of the processed BAM files.

ls Nuc_clipped_dedup_RG_Filtered_Sorted_*_PA42.4.2_mtDNA.bam > Nuc_clipped_BAMs_TP1.txt

b. Run the mpileup function of Samtools.

samtools mpileup -f /home/tmaruki/PA42.4.2_mtDNA/PA42.4.2_mtDNA.fasta -b Nuc_clipped_BAMs_TP1.txt -o Nuc_clipped_14Lines_TP1_PA42.4.2.mpileup

2. Extract the nucleotide at every position of the reference sequence using Ext_Ref_Nuc.pl.

perl Ext_Ref_Nuc.pl PA42.4.2.fasta.masked RefNuc_PA42.4.2.masked.txt

3. Make a pro file of nucleotide read counts from the mpileup file using mpileup2pro.

./mpileup2pro -ref RefNuc_PA42.4.2.masked.txt -id IDs_TP1.txt -mp Nuc_clipped_14Lines_TP1_PA42.4.2.mpileup -out Nuc_clipped_14Lines_TP1_PA42.4.2.pro

4. Run HGC to identify tri- and tetra-allelic sites, setting the minimum required coverage to call individual genotypes at six.

./HGC -in Nuc_clipped_14Lines_TP1_PA42.4.2.pro -min_cov 6 -out Out_mc6_HGC_14Lines_TP1_PA42.4.2.txt

5. Set the depth of coverage of all individuals to zero in the pro file at tri- and tetra-allelic sites using Rem_Multi_Allelic.

a. Make a list of coordinates of the tri- and tetra-allelic sites.

awk -v OFS='\t' '{if ($1 == "scaffold" || $4 != "NA" && $4 > 2) print $1, $2}' Out_mc6_HGC_14Lines_TP1_PA42.4.2.txt > List_MA_Out_mc6_HGC_14Lines_TP1_PA42.4.2.txt

b. Run Rem_Multi_Allelic.

./Rem_Multi_Allelic -pf Nuc_clipped_14Lines_TP1_PA42.4.2.pro -mf List_MA_Out_mc6_HGC_14Lines_TP1_PA42.4.2.txt -out MAR_Nuc_clipped_14Lines_TP1_PA42.4.2.pro

6. Run GFE_v3.0 in the c mode.

./GFE_v3.0 -in MAR_Nuc_clipped_14Lines_TP1_PA42.4.2.pro -mode c -out Out_c_GFEv3.0_14Lines_TP1_PA42.4.2.txt

7. Run bmf_v1.0, setting the minimum required coverage to call individual genotypes at eight and outputting reference nucleotides.

./bmf_v1.0 -in Out_c_GFEv3.0_14Lines_TP1_PA42.4.2.txt -rn 1 -min_cov 8 -out Out_rn1_mc8_bmfv1.0_14Lines_TP1_PA42.4.2.txt

8. Apply the total-coverage, error-rate estimate, and repeat filters to the bmf output.

awk '{if ($1 == "scaffold" || $3 !~ /[acgtn]/ && $4 <= 0.01 && $5 >= 40 && $5 <= 400) print}' Out_rn1_mc8_bmfv1.0_14Lines_TP1_PA42.4.2.txt > NR_EF_DCF_Out_rn1_mc8_bmfv1.0_14Lines_TP1_PA42.4.2.txt
