# Supplementary material for: What can we infer about mutation calling by using time‐series mutation accumulation data and a Bayesian Mutation Finder?
Source: Ecol Evol. 2024 Nov 10;14(11):e70339. doi: 10.1002/ece3.70339 (PMC11550904; doi:10.1002/ece3.70339)
Supplement: Supplementary file 15 — Text S3 [file ECE3-14-e70339-s001.docx]

Supporting Information for:

What can we infer about mutation calling by using time-series mutation accumulation data and a Bayesian Mutation Finder?

Takahiro Maruki, April Ozere, Jack Freeman, and Melania E. Cristescu

**Text S3** GATK protocol.

1. Call variants using HaplotypeCaller in GATK, outputting an indel-realigned BAM file.

gatk HaplotypeCaller \

-R PA42.4.2_mtDNA.fasta \

-I Nuc_clipped_dedup_RG_Filtered_Sorted_C001-88_PA42.4.2_mtDNA.bam \

-O Nuc_C001-88_PA42.4.2_mtDNA.g.vcf.gz \

-ERC GVCF \

-bamout Realigned_Nuc_clipped_dedup_RG_Filtered_Sorted_C001-88_PA42.4.2_mtDNA.bam

2. Run GenomicsDBImport of GATK.

gatk GenomicsDBImport \

--genomicsdb-workspace-path TimePoint1_database \

-L scaffolds_PA42.4.2.list \

--sample-name-map TimePoint1_map \

--tmp-dir tmp_GATK

3. Run GenotypeGVCFs of GATK

gatk GenotypeGVCFs \

-R PA42.4.2_mtDNA.fasta \

-V gendb://TimePoint1_database \

-O TimePoint1.vcf.gz \

--tmp-dir tmp_GATK

4. Select SNPs from the VCF file excluding sites involved in repetitive regions, using SelectVariants in GATK.

gatk SelectVariants \

-V TimePoint1.vcf.gz \

-select-type SNP \

-XL PA42.4.2_repeats.list \

-O SNPs_TimePoint1.vcf.gz

5. Apply the hard filtering to the SNPs, using VariantFiltration in GATK.

gatk VariantFiltration \

-V SNPs_TimePoint1.vcf.gz \

-filter "QD < 2.0" --filter-name "QD2" \

-filter "QUAL < 30.0" --filter-name "QUAL30" \

-filter "SOR > 3.0" --filter-name "SOR3" \

-filter "FS > 60.0" --filter-name "FS60" \

-filter "MQ < 40.0" --filter-name "MQ40" \

-filter "MQRankSum < -12.5" --filter-name "MQRankSum-12.5" \

-filter "ReadPosRankSum < -8.0" --filter-name "ReadPosRankSum-8" \

-O Filtered_SNPs_TimePoint1.vcf.gz

6. Extract necessary fields from the VCF file of the hard-filtered SNPs, using VariantsToTable in GATK.

gatk VariantsToTable \

-V Filtered_SNPs_TimePoint1.vcf.gz \

-F CHROM -F POS -F REF -F ALT -F QUAL -F FILTER -F AC -F AN -F DP -GF GT -GF AD -GF DP -GF GQ \

-O Filtered_SNPs_TimePoint1.table
