# Supplementary material for: What can we infer about mutation calling by using time‐series mutation accumulation data and a Bayesian Mutation Finder?
Source: Ecol Evol. 2024 Nov 10;14(11):e70339. doi: 10.1002/ece3.70339 (PMC11550904; doi:10.1002/ece3.70339)
Supplement: Supplementary file 16 — Text S4 [file ECE3-14-e70339-s014.docx]

Supporting Information for:

What can we infer about mutation calling by using time-series mutation accumulation data and a Bayesian Mutation Finder?

Takahiro Maruki, April Ozere, Jack Freeman, and Melania E. Cristescu

**Text S4** Literature-informed rationale for parameter cutoff values used for GATK hard filtering.

QD < 10

Supporting Literature: According to Latorre *et al.* (2023), QD discriminates false calls better than all other summary statistics. They set the QD cutoff at one SD from the median. Data from Koch *et al.* (2019) also supported a higher QD cutoff; they noted that variants with QD < 4 had a tendency to be false positive calls. Despite the recommendation of Lefouili and Nam (2022), to use Variant Quality Score Recalibration (VQSR) due to the bimodal QD distribution (Figure S2), we chose an approach that emphasized hard filtering because according to experts, VQSR is not suitable for *de novo* mutation experiments due to the tendency to remove *de novo* variants (Bergeron *et al.* 2021). We chose a QD cutoff of < 10 because we determined that a high QD cutoff would eliminate a significant portion of suspected false positive calls. Despite that most surveyed literature used the GATK general cutoff of QD < 2 (Table S2) (Van der Auwera *et al.* 2013), we chose a higher cutoff because of the effectiveness of QD filtering (Latorre *et al.* 2023).

MQ < 40

Supporting Literature: According to Lefouili and Nam (2022), when QD is bimodal (Figure S2), VQSR is the only reliable pipeline. However, according to experts, VQSR is not suitable for *de novo* mutation experiments and hard filtering should be employed instead (Bergeron *et al.* 2022). Faced with this dilemma, we chose the most widely used cutoff in surveyed literature (Table S2), which was also supported by *de novo* mutation calling experts Bergeron *et al.* (2021) notwithstanding adjustments made to other filters to reduce the false positive rate.

SOR > 3

Supporting Literature: SOR > 3 was the most widely used and most stringent SOR cutoff in surveyed literature (Table S2). Nevertheless, many studies omitted this parameter (Table S2). Despite this, we employed the SOR cutoff to filter low quality variants.

FS > 60

Supporting Literature: We chose this cutoff because it was the most widely used FS cutoff in surveyed mutation literature (Table S2), and we had employed more stringent cutoffs for other parameters. As such we were cautious about the potential of stringent filtering to increase the incidence of false negatives (Bergeron *et al.* 2022).

MQ Rank Sum < -2

Supporting Literature: *De novo* mutation calling experts, Bergeron *et al.* (2021) examined parameter values apparent in false positive calls and adjusted the hard filtering cutoffs accordingly to reduce the incidence of false positives. As such, they set the MQ Rank Sum cutoff at < –2. Another *de novo* mutation analysis, Latorre *et al.* (2020), also set the same cutoff for MQ Rank Sum (< -2). Both groups of researchers also set an upper cutoff (4 and 2, respectively). Koch *et al.* (2019) also noted that variants with MQ Rank Sum < -2 had a tendency to be false positive calls. Given the support for the MQ Rank Sum < -2 cutoff, we chose to employ it, but we did not specify an upper bound as we were cautious of the effect of overly stringent filtering on the false negative rate (Bergeron *et al.* 2022).

Read Pos Rank Sum < -3.5

Supporting Literature: We employed a similar cutoff to those used by *de novo* mutation calling experts: Bergeron *et al*. (2021) (-3), Latorre *et al.* (2020) (-2), and Bergeron *et al.* (2022) (-2.5 and –3) (see “Comments” in Table S2 for an explanation of the use of multiple hard filtering cutoffs). Though these researchers also employed upper cutoffs, equal to the absolute value of the lower cutoff (Table S2), we did not employ an upper cutoff because we sought to avoid overly stringent hard filtering.

**References**

Assaf, Z. J., S. Tilk, J. Park, M. L. Siegal and D. A. Petrov, 2017 Deep sequencing of natural and experimental populations of *Drosophila melanogaster* reveals biases in the spectrum of new mutations. Genome Res 27: 1988-2000.

Balik-Meisner, M., L. Truong, E. H. Scholl, R. L. Tanguay and D. M. Reif, 2018 Population genetic diversity in zebrafish lines. Mamm Genome 29: 90–100.

Becker, D., K. Barnard-Kubow, R. Porter, A. Edwards, E. Voss *et al.,* 2022 Adaptive phenotypic plasticity is under stabilizing selection in *Daphnia*. Nat Ecol Evol 6: 1449–1457.

Bergeron, L. A., S. Besenbacher, J. Bakker, J. Zheng, P. Li *et al.,* 2021 The germline mutational process in rhesus macaque and its implications for phylogenetic dating. GigaScience 10: giab029.

Koch, E. M., R. M. Schweizer, T. M. Schweizer, D. R. Stahler, D. W. Smith *et al.,* 2019 De novo mutation rate estimation in wolves of known pedigree. Mol Biol Evol 36: 2536–2547.

Kurta, K., H. Jeuthe, R. Naboulsi, D. de Koning and C. Palaiokostas, 2023 Seasonal and age-related changes in sperm quality of farmed arctic charr (*Salvelinus alpinus*). BMC Genomics 24: 519.

Latorre, S. M., C. S. Reyes-Avila, A. Malmgren, J. Win, S. Kamoun *et al.,* 2020 Differential loss of effector genes in three recently expanded pandemic clonal lineages of the rice blast fungus. BMC Biol 18: 88.

Latorre, S. M., V. M. Were, A. J. Foster, T. Langner, A. Malmgren *et al.,* 2023 Genomic surveillance uncovers a pandemic clonal lineage of the wheat blast fungus. PLoS Biol 21: e3002052.

Malo, M. E., Z. Schultzhaus, C. Frank, J. Romsdahl, Z. Wang *et al.,* 2021 Transcriptomic and genomic changes associated with radioadaptation in *Exophiala dermatitidis*. CSBJ 19: 196-205.

Thomas, G. W. C., R. J. Wang, A. Puri, R. A. Harris, M. Raveendran *et al.,* 2018 Reproductive longevity predicts mutation rates in primates. Curr Biol 28: 3193-3197 e3195.

Wu, F. L., A. I. Strand, L. A. Cox, C. Ober, J. D. Wall *et al.,* 2020 A comparison of humans and baboons suggests germline mutation rates do not track cell divisions. PLoS Biol 18: e3000838.
